# Mental Health, Burnout, and Job Stressors Among Healthcare Workers During the COVID-19 Pandemic in Iran: A Cross-Sectional Survey

**DOI:** 10.3389/fpsyt.2022.891430

**Published:** 2022-05-12

**Authors:** Ahmad Hajebi, Maryam Abbasinejad, Masoud Zafar, Amirali Hajebi, Farhad Taremian

**Affiliations:** ^1^Psychiatric Department, Research Center for Addiction and Risky Behaviors, Iran University of Medical Sciences, Tehran, Iran; ^2^Department for Mental Health and Substance Abuse, Ministry of Health and Medical Education, Tehran, Iran; ^3^University Counselling Center, Shahed University, Tehran, Iran; ^4^Non-communicable Diseases Research Center, Endocrinology and Metabolism Research Institute, Tehran University of Medical Sciences, Tehran, Iran; ^5^Department of Clinical Psychology, University of Social Welfare and Rehabilitation Sciences, Tehran, Iran

**Keywords:** mental health, burnout, job stressors, COVID-19, healthcare workers, Iran

## Abstract

**Introduction:**

The COVID-19 pandemic has caused increasing levels of mental health problems such as anxiety and depression among doctors, nurses and other healthcare workers in hospitals or health centers. The main objective of this study was to assess the mental health, job stressors, and burnout among healthcare workers in Iran.

**Materials and Methods:**

A cross-sectional study was performed in the primary healthcare centers and hospitals affiliated with six of the medical universities in Iran. The selection of participants was done using multi-center convenient sampling. The Patient Health Questionnaire-9, Generalized Anxiety Disorder-7, and Copenhagen Burnout Inventory were used for gathering data through an online platform. Data related to job stressors were obtained using a validated checklist. Data analysis was performed using Chi-square and multiple regression tests and the phi coefficient.

**Results:**

The results of our study showed that 53% of the healthcare workers of the hospitals and primary healthcare centers enrolled in our study either had generalized anxiety disorder or major depressive disorder or both disorders. Moderate and high levels of burnout were seen among 48.9% of the study participants. The prevalence of mental disorders and burnout were significantly higher among the female healthcare workers compared to the male (*p* = 0.0001) and a higher rate of mental disorder and burnout was also seen among healthcare workers of hospitals compared to those working in primary healthcare centers (*p* = 0.024). “Worry about children and old members of family,” “family worries for my health condition” and “lack of specific effective treatment for COVID-19” were found to be predictive of mental disorder and burnout. The most prevalent job stressor among the total sample was “low payment or income during the COVID-19 period”.

**Conclusion:**

The results of our study revealed high psychological distress and burnout among healthcare workers of the hospitals during the fourth peak of the COVID-19 pandemic in Iran. This study highlights the need for health officials to pay attention to the job stressors of healthcare workers and obliges them to perform effective interventions to address their needs and concerns.

## Introduction

The World Health Organization (WHO) declared the SARS-Cov-2 outbreak as a pandemic on March 11th 2020 (1). Until March 2022, which is the time of inscribing this article, the COVID-19 cases worldwide have surpassed 445 million and there have been more than 6 million COVID-19 reported deaths confirmed globally (2). The uncontrollable nature of COVID-19 has posed remarkable challenges to the health care systems in most of the nations affected (3). Some countries have reported temporary shortage of health care providers and/or health equipment and supply in the midst of the peaks of the pandemic (4). Other than that, the current pandemic has caused increasing levels of mental health problems such as anxiety and depression among doctors, nurses and other healthcare workers in hospitals or health centers (5). As reported, many health personnel have lost their lives due to COVID-19 and many of them have been infected with the virus or transferred the illness to their family members (6).

Pandemics such as the COVID-19 provoke fear and anxiety, which is common among healthcare workers who are directly involved in the management of ill patients. The healthcare workers' exposure to patients' suffering and deaths (7) also increases their fear and anxiety. Unattended anxiety negatively impacts work performance and job satisfaction of health personnel, leading to frequent absenteeism and eventual turnover (8). Available data suggest that the prevalence of anxiety and depression among health care workers during the COVID-19 pandemic ranged from 22.6% (9) to 47% (10) and 22.8% (7) to 50.4% (11) respectively. The prevalence of burnout among healthcare workers in the COVID-19 has also been frequently studied in different regions and countries and high-level burnout rates of 49.3% (12) and 50% (13), to more than 60% (14) and even 67% (15) have been reported among healthcare workers in the COVID-19 pandemic. Studies have also shown high rates of anxiety and depression among Iranian healthcare workers in the COVID-19 pandemic (16–18), especially among the female (19). Iranian studies have shown negative psychological experiences caused by COVID-19 in healthcare workers, such as fatigue, discomfort, and helplessness due to high-intensity work, anxiety, and worry about family members (20). An Iranian study performed by Jalili et al. (21) have reported high levels of burnout among 53% of the healthcare workers in the COVID-19 pandemic.

Studies have defined variable psychosocial stressors that the COVID-19 pandemic has brought about and led to exceeding levels of psychological distress (22), mental health problems such as anxiety and depression (23), and burnout (5, 24–26) among healthcare personnel worldwide.

Throughout the COVID-19 pandemic, frontline healthcare workers have been facing the fear of infection of themselves or their families and dealt with considerable initial uncertainty about disease standard treatment regimens (27). They have also been faced with complex ethical issues in practice, and frustrated by moral conflicts (28). Excessive workloads, long working hours, lack of enough time for recovery, insufficient personal protective equipment at the beginning of the pandemic, not being able to tell their manager if they are not coping (29), and inadequate hospital facilities for the patients are all important factors that have put them under persistent pressure and sometimes affected the quality of patient care (10).

Several psychosocial and demographic variables like gender, age, profession, place of work, family income (30), and risk factors such as poor social support, low senses of self-efficacy, and experiencing stigma (22) are associated with increased stress, anxiety and depressive symptoms and burnout (31) among healthcare workers during the COVID-19 pandemic (32). For instance, some studies convey that being a woman (33), having a younger age, being the parents of dependent children (34), and working in high-risk areas may have more negative psychological health outcomes (11). This reality is even more negative in the case of nurses due to the high emotional burden of continued contact with patients' suffering and pain (35).

The main objective of this study was to assess the prevalence of depression, anxiety and burnout among Iranian healthcare workers of the PHC system and hospitals in the COVID-19 pandemic. This is the first large-scale multi-provincial study performed for assessing burnout and adverse mental health effects of the COVID-19 pandemic on a heterogeneous group of healthcare workers in Iran.

## Materials and Methods

### Study Design and Settings

A cross-sectional study was performed in the primary healthcare centers and hospitals affiliated with six medical universities located in the provinces of Tehran, Tabriz, Gilan, Ahvaz, Ghom, and Kurdistan of Iran. The medical universities were selected based on their geographical diversity and the capacity of their primary healthcare centers for providing services. In each university, one hospital and five primary healthcare centers were chosen for performing the study.

### Study Participants

The target population in this study included physicians, nurses, dentists, mental health workers, environmental and occupational health workers, community health workers, medical technicians, and staff members working in hospitals and primary healthcare centers. The samples size was calculated to be 1,055. We considered a sample size which was 10% larger to cover possible drop-out of participants and ultimately came up with an approximate number of 1,170 participant. According to probability-proportional-to-size sampling (36), 120 individuals were selected from each hospital, making a total of 720 participants from the hospital setting and 15 individuals were selected for each PHC center, making a total of 450 participants from primary healthcare centers to cover different job categories. The selection of participants was done using multi-center convenient sampling. Any healthcare worker working at hospitals or PHC centers at the time of the study, working at least between 44 and 50 h a week was recognized eligible to receive the questionnaire link and was asked to respond to all of the questions in the time period announced, and therefore was initially enrolled in the study. Only one reminder was performed 2 weeks after the initial call for participation. At the end of the data gathering process, 37 participants who had missing demographic data or had not filled out one or more of the questionnaires were excluded from the study and 1,133 participants remained.

### Study Instruments

Demographic data were obtained in the beginning of the questionnaire. Data related to job stressors were obtained using a checklist which was developed after thorough literature and desk review and finalized after performing individual deep interviews with experts and focus group discussions (FGD) with members of each of the target groups. We initially came up with a job stress checklist of 80 items. Content validity of all items was measured with the consultation of 10 experts and items with a CVR of lower than 0.75 (37) and an I-CVI of lower than 0.78 (38) were excluded from the checklist, and so the final checklist was consisted of 65 items. For identifying the top ten stressor as selected by the participants, all of the 65 stressors were presented to the participants in the online questionnaire and participants were asked to score the importance of each stressor on a 5-point Likert scale.

Data related to the study variables, burnout, and mental health status were obtained using online validated questionnaires, as follows:

#### Patient Health Questionnaire-9

The PHQ-9 (39) is a nine-item instrument designed for detecting major depressive disorder (MDD) based on the fourth version of the Diagnostic and Statistical Manual of Mental disorders (DSM-IV) (40). The internal reliability of the PHQ-9 was excellent, with a Cronbach's α of 0.89 in the PHQ Primary Care Study (39). Scores are calculated based on how frequently a person experiences the mentioned feelings. In scoring, each “not at all” response is scored as 0; each “several days” response is 1; each “more than half the days” response is 2; and each “nearly every day” response is 3 (39). Therefore, scores range from 0 to 27 with higher scores indicating more severe MDD symptoms. The PHQ-9 has been validated for use among the Iranian population with a Cronbach's α of 0.856 (41) and a cut-off score of 13 which provided an optimal balance between sensitivity and specificity (42).

#### Generalized Anxiety Disorder-7

The GAD-7 (43) is a seven-item self-report scale developed for the diagnosis of generalized anxiety disorder (GAD) according to the DSM-IV. The GAD-7 score is calculated by assigning scores of 0, 1, 2, and 3, to the response categories of “not at all,” “several days,” “more than half the days,” and “nearly every day”. Scores range from 0 to 21 with higher scores indicating more severe GAD symptoms (43). The GAD-7 has been validated to use among the Iranian population with an α Cronbach value of 0.88 and a cut-off point of 10 for diagnosing GAD (44).

#### Copenhagen Burnout Inventory

The CBI (45) was used to measure the fatigue and exhaustion as core constructs of burnout among participants. This 19-item questionnaire measures three burnout sub-dimensions: personal burnout (6 items), work-related burnout (7 items), and client-related burnout (6 items). Each item is scored by the participant on a 5-point Likert scale. Scores fall into the four categories of “no burnout” (0–25), “mild burnout” (25.1–50), “moderate burnout” (50.1–75), “high burnout” (75.1–99). Separate scores can be reported for each sub-dimension and the total score of the test is the mean score of all three sub-dimensions (46). The original version of the instrument had presented a good internal consistency for all three subscales: personal burnout (α = 0.87), work related burnout (α = 0.87), and client-related burnout (α = 0.85). The internal consistency of the Persian version ranged from a Cronbach's α of 0.82 to 0.90 and the test-retest reliability was excellent with the ICC ranging from 0.85 to 0.95 (47).

### Procedure

In each university, the director of the mental health department was the coordinator of the project. Project managers were appointed for each of the hospitals for coordination and monitoring of the process of data collection. The project coordinators and managers were trained through a 1-day training session and were instructed on the sampling method, research design and assessment tools. A detailed guideline on the aim of the study and the methodology including number of participants needed for each job category were also sent to them via electronic mail. Participants were chosen by the project coordinator and project managers by convenient sampling. The PORS-LINE platform was used for gathering data. The link of the questionnaire was sent to each participant and they were asked to fill out the questionnaire from June 23rd 2021 to July 18th 2021. It is worth mentioning that this study was performed in the fourth peak of COVID-19 in Iran which was simultaneous with the peaks in India and Brazil (48).

### Data Analysis

Data entry and analysis was performed with the SPSS V.23 software. We used descriptive analysis using one and two-variable frequency tables for displaying numbers, percentages and frequencies. Chi-square tests were used for qualitative variables, and the phi coefficient was used for two-state variables and multiple regression tests were used for continuous quantitative variables.

## Results

### Demographic Data

At the end of the deadline of data gathering, 37 questionnaires were excluded from the study because the participant had not filled out some of the questionnaires, and we ultimately had 1,133 completed questionnaires from 1,133 participants. Among these, 755 (66.6%) were female. The majority (66.5%) of the participants were aged 20 to 40 and were married (73.3%). Participants were consisted of physicians, nurses and other workers and staff members from which 715 (63.1%) of them worked at hospitals. The largest group regarding occupation were the nurses with 41 percent of the participants (see Table 1).

**Table 1:** Demographic data of the study sample (*n* = 1,133).

**Variable**		**Frequency (percent)**
Gender	Male	378 (33.4)
	Female	755 (66.6)
Marital status	Single	276 (24.4)
	Married	830 (73.3)
	Widowed	5 (0.4)
	Divorced	22 (1.9)
Age	20–30	301 (26.6)
	31–40	452 (39.9)
	41–50	287 (25.2)
	51–60	90 (7.9)
	61 <	3 (0.3)
Occupation type	Physicians	244 (21.9)
	Nurses	463 (40.9)
	Medical technicians	107 (9.4)
	Administrative staff	96 (8.5)
	Service staff	108 (9.5)
	Dentists	28 (2.5)
	Mental health workers	44 (3.9)
	Environmental and occupational health workers	43 (3.8)
	Total	1,133 (100)

### Job Stressors

The most prevalent job stressor among the total sample was “low payment or income during the COVID-19 period,” and “worry about family members being infected by COVID-19,” “worry about my transmitting COVID-19 to family members,” and “worry about children and old members of the family” came afterwards (see Table 2).

**Table 2:** Top 10 ranks of reported stressors among total sample (*n* = 1,133).

**Stressors**	**Total rank**
Low payment and income in these days	1
Worry about family members being infected by COVID-19	2
Worry about my transmitting COVID-19 to family members	3
Worry about children and old members of the family	4
Lack of special payment or low payment for the COVID-19 period	5
The Ministry of Health not keeping their promises	6
No payments for overtimes	7
Family worries for my health condition	8
Low support of authorities of the Ministry of Health	9
Lack of a specific effective treatment for COVID-19	10

### Mental Disorder

The presence of generalized anxiety disorder (GAD) and major depressive disorder (MDD) among study participants was assessed with the PHQ-9 and GAD-7 tools respectively. According to the results, among the male participants, 35.7 and 30.2% and among the female participants, 53.1 and 38.9% had GAD and MDD respectively. Considering both genders, among the 1,133 participants, a total of 536 (47.3%) participants had GAD and 408 (36%) had MDD, and a total of 600 (53%) of the participants had either GAD or MDD or both of the disorders, which is referred to as “any mental disorder.” Among those with any mental disorder, 344 (30.36%) had both MDD and GAD, 192 (16.9%) had only GAD and 64 (5.6%) had only MDD. Study results showed that 58.2% of the female and 42.6% of the male participants had any mental disorder. A higher rate of mental disorder among the female compared to the male participants was statistically significant (*p* = 0.0001) (see Table 3).

**Table 3:** Frequency and percentage of any mental disorder by gender (*n* = 1,133).

**Gender**	**Mental health**		**Total**
	**No mental disorder**	**Any mental disorder**	
	***N* (%)**	***N* (%)**	
Male	217 (57.4)	161 (42.6)	378 (100)
Female	316 (41.8)	439 (58.2)	755 (100)
Total	533 (47)	600 (53)	1,133 (100)

*Phi = 0.147 (p = 0.0001)*.

Regarding workplace, a total of 397 participants (55.5%) of those working in hospitals and 203 (48.6%) of those working in PHC centers had any mental disorder. The higher rate of mental disorders of the healthcare workers in the hospitals was statistically significant (*p* = 0.024).

### Burnout

According to the results, among the 1,133 participants, a total of 554 (48.9%) participants had moderate and high level burnout (36%). Also, study results showed that 381 (50.5%) of the female and 173 (45.8%) of the male participants had moderate and high level burnout. A higher rate of burnout among the female compared to the male participants was statistically significant (*p* = 0.001) (see Table 4). The mean score of burnout among individuals was 61.1 in the participants with any mental disorder and 37.7 in the participants without any mental disorder. This means that there is a significant relationship between the presence of anxiety and depression with burnout (*p* < 0.0001, t = 21.054).

**Table 4:** Frequency and percentage of burnout by gender (*n* = 1,133).

**Gender**	**Burnout**				**Total**
	**No burnout (CBI: 0–25)**	**Low level burnout (CBI: 26–50)**	**Moderate level burnout (CBI: 51–75)**	**High level burnout (CBI: 76–100)**	
	***N* (%)**	***N* (%)**	***N* (%)**	***N* (%)**	***N* (%)**
Male	80 (21.2)	125 (33.1)	119 (31.5)	54 (14.3)	378 (100)
Female	92 (12.2)	282 (37.4)	255 (33.8)	126 (16.7)	755 (100)
Total	172 (15.2)	407 (35.9)	374 (33)	180 (15.9)	1,133 (100)

*Pearson Chi-Square = 15.97 (p-value = 0.001)*.

Regarding workplace, a total of 375 participants (52.4%) of those working in hospitals and 179 (42.8%) of those working in PHC centers had moderate to high levels of burnout. The higher rate of burnout of the healthcare workers in the hospitals in comparison to the PHC centers was statistically significant (*p* = 0.011). Our findings showed a significant correlation between burnout and MDD (R = 0.64, *p* = 0.0001) and also between burnout and GAD (R = 0.58, *p* = 0.0001).

### Predictors of Mental Disorder and Burnout

The regression performed for predicting any mental disorder based on the ten top-ranking stressors showed that the regression coefficient is equal to 0.374 and the determination coefficient (R^2^) is equal to 0.14. In other words, the ten stressors predicted 14 percent of any mental disorder. The F is equal to 18.27 and confirms the significance of the regression model with a 0.01 error (see Table 5).

**Table 5:** Multiple regression model for predicting any mental disorder among the study sample.

	**B (Beta)**	**Std. error**	**t (P v)**
(Constant)	−0.479	0.084	−5.679 (0.0001)
Low payment and income in these days	0.022 (0.046)	0.018	1.208 (0.227)
Worry about family members getting COVID-19	−0.003 (−0.006)	0.015	−0.189 (0.85)
Worry about my family members getting COVID-19 by me	0.013 (0.033)	0.014	0.990 (0.32)
**Worry about children and old members of family**	**0.068 (0.148)**	**0.017**	**4.137 (0.0001)**
Lack of special payment or low payment for COVID-19 days	0.015 (0.034)	0.018	0.845 (0.398)
Not keeping promises given by Ministry of Health	0.000 (−0.001)	0.017	−0.014 (0.99)
No payments for overtimes	0.001 (0.001)	0.015	0.039 (0.97)
**Family worries for my health condition**	**0.039 (0.089)**	**0.014**	**2.781 (0.006)**
Low support of authorities of Ministry of Health	0.027 (0.069)	0.016	1.736 (0.083)
**Lack of specific effective treatment for COVID-19 disease**	**0.052 (0.129)**	**0.015**	**3.568 (0.0001)**

*Dependent variable: Any mental disorders. The bold values indicate significantly predict the dependent variables*.

The regression model shows that “worry about children and old members of family,” “family worries for my health condition,” “lack of specific effective treatment for COVID-19” could significantly predict the outcome of any mental disorder.

The regression performed for predicting burnout based on the ten top-rank stressors showed that the regression coefficient (R) was 0.437 and the determination coefficient was (R^2^) 0.19. Therefore, the top ten stressors were able to predict 19 percent of the outcome of burnout. The F value is equal to 26.55 and it confirms the significance of the regression model with an error of 0.01 (see Table 6).

**Table 6:** Multiple regression model for predicting burnout among the study sample.

	**B (Beta)**	**SE**	**t (P v)**
(Constant)	−2.736	3.633	−0.753 (0.452)
**Low payment and income in these days**	**1.490 (0.071)**	**0.778**	**1.915 (0.056)**
Worry about family members getting COVID-19	0.438 (0.023)	0.640	0.684 (0.494)
Worry about my family members getting COVID-19 by me	−0.426 (−0.024)	0.585	−0.729 (0.466)
**Worry about children and old members of family**	**2.258 (0.110)**	**0.712**	**3.173 (0.002)**
**Lack of special payment or low payment for COVID-19 days**	**1.436 (0.073)**	**0.758**	**1.894 (0.059)**
**Not keeping promises given by Ministry of Health**	**1.566 (0.085)**	**0.735**	**2.131 (0.033)**
No payments for overtimes	−0.062 (−0.003)	0.648	−0.095 (0.924)
**Family worries for my health condition**	**1.841 (0.096)**	**0.596**	**3.087 (0.002)**
Low support of authorities of Ministry of Health	1.071 (0.062)	0.673	1.590 (0.112)
**Lack of specific effective treatment for COVID-19 disease**	**2.632 (0.147)**	**0.626**	**4.206 (0.0001)**

*Dependent variable: Burnout. The bold values indicate significantly predict the dependent variables*.

The regression model shows that among the ten top-ranking stressors, “low payment and income,” “worry about children and old members of the family,” “lack of special payment or low payment for COVID-19 days,” “the MOH not keeping their promises,” “family worries for the individuals' health condition,” and “the lack of specific effective treatment for COVID-19” could predict burnout significantly.

For summarizing the results of the multiple regression models performed for assessing the relationship between the top ten stressors with the outcomes of any mental disorder and burnout, Table 7 is presented. In this table, the job stressors which could significantly predict the outcomes are bolded. As is seen, “worry about children and old members of family,” “family worries for my health condition” and “lack of specific effective treatment for COVID-19” are the three job stressors that could significantly predict both of the two outcomes of mental disorder and burnout (see Table 7).

**Table 7:** Summary of multiple regression analysis for top ten stressors predicting dependent variables.

	**Dependent variables**	
**Top ten stressors**	**Burnout**	**Mental disorder**
**Low payment and income in these days**	•	
Worry about family members getting COVID-19		
Worry about my family members getting COVID-19 by me		
**Worry about children and old members of family**	•	•
**Lack of special payment or low payment for COVID-19 days**	•	
**Not keeping promises given by Ministry of Health**	•	
No payments for overtimes		
**Family worries for my health condition**	•	•
Low support of authorities of Ministry of Health		
**Lack of specific effective treatment for COVID-19 disease**	•	•

*The bold values indicate significantly predict the dependent variables*.

## Discussion

Our study shows a higher than 50% prevalence of mental disorders among healthcare workers of hospitals and PHC centers in the midst of one of the deadliest COVID-19 pandemic peaks in Iran. This reported prevalence is higher than that of the general population which has been reported to be 29.7% in the same time period (49). This finding is similar to a number of international (7, 9–11) and national (16–20) studies conducted at the time of the COVID-19 pandemic for assessing psychological distress and mental health problems among healthcare workers which have reported a high prevalence of anxiety and depression. One similar study performed a year earlier among healthcare workers from twelve different cities in India revealed that 52.9% of the participants had the risk of psychological distress that needed further evaluation (50). The higher prevalence of mental disorders among female healthcare workers found in our study has also been replicated in many studies (51–55). Results also showed a raised prevalence ratio of female/male regarding GAD and a decreased prevalence ratio of female/male regarding MDD among the healthcare workers compared to previous population studies (56).

Moderate to severe levels of burnout have been seen among nearly half of our study participants, mostly reported in healthcare workers of hospitals. Our findings are similar to findings of other studies assessing burnout levels among health care workers in the COVID-19 pandemic (12–14), one study even showing a burnout prevalence of 67% (15). Alrawashdeh et al. (33) showed in their study which was performed among physicians that several significant factors were positively associated with burnout, including female gender, working at highly loaded hospitals, working for long hours, doing night shifts, and lack of sufficient access to personal protective equipment. Regarding the fact that the further mentioned risk factors for burnout are usually mostly relevant to hospitals and not outpatient centers, we can perhaps conclude that the higher prevalence of burnout in hospitals found in our study can be rationalized. Results of one study showed a significant association of depression, anxiety and stress with cumulative psychological burnout, consistent with our study results (57).

The most prevalent job stressor reported by the participants of our study was low payment and income in the COVID-19 days. Concerning the role of the sufficiency of the income for family needs, Tarcan et al. (58) suggest that higher income tended to be related with better health status and with lower burnout levels, both for general physicians and nurses. Several authors have identified a relationship between better payment or income earned and higher job satisfaction, which in turn decreases burnout level (59–61). Despite this general view, one study (62) did not find such relation between payment and burnout syndrome and Linzer et al. (63) even concluded that the relevant factor for physicians was rather the relationship with patients than the monetary compensation.

Our study findings have shown that the three factors of “worry about children and old members of the family,” “family worries for my health condition,” and “lack of a specific effective treatment for COVID-19” can significantly predict the severity of the two outcomes of mental disorder and burnout. Concerns about personal and family health (64) and fear for personal and family safety had (65) also been reported among healthcare workers throughout different epidemics such as the SARS and MERS. Worry about children among the healthcare workers in the COVID-19 pandemic has been stated in other studies, especially among nurses who have been distanced from their children for a while (66). One study in the United States also showed that it was most stressful for healthcare workers to think they could transmit the disease to their family and friends (67). This finding has also been replicated in many other studies (8, 10, 23). Results of other studies have also revealed more psychological stress among married healthcare workers and those having children (68, 69), possibly resembling the concern these individuals have about the health of their family members.

Conducting this study during the ongoing outbreak of COVID-19 and in the midst of one of the worst pandemic peaks in Iran imposed methodological limitations for our study. The main one was that an online platform was selected for gathering data from the participants in order to minimize human encountering. This prevented the researchers to select a specific time, place and circumstance for meeting the participants and filling out the questionnaires, which could have enhanced the quality of the data gathering. Convenient sampling also limits the generalizability of our results due to possible under-representation of the study sample as a whole.

One of the main strength points of this study is that the researchers managed to reach out to a great number of healthcare workers in six provinces in different geographical zones in the country. Another strength point was that a holistic approach was adopted and different types of healthcare workers and health staff who worked in hospitals and PHC centers were selected for enrollment in the study. Standard tools have been used for assessing mental disorders and burnout among the participants in this study which enables comparison with the results of other studies of this kind.

## Conclusion

The results of our study revealed high psychological distress and burnout among healthcare workers of the hospitals during the fourth peak of the COVID-19 pandemic in Iran. Higher levels of distress among female healthcare workers and those working in hospitals as seen in many other studies should be an area of special consideration and special interventions should be conducted in this regard. Burnout among healthcare workers may lead to lower quality of care for the patients and increase the probability of medical mismanagement, which is a critical issue regarding the fact that the health system is faced with shortage of human resources and high workload due to the COVID-19 pandemic. This study highlights the need for health officials to pay attention to the stressing effect of low payment and also the fear and concerns of healthcare workers about personal and family safety in the COVID-19 pandemic and obliges them to perform effective interventions to address their needs and concerns.

## Data Availability Statement

The raw data supporting the conclusions of this article will be made available by the authors, without undue reservation.

## Ethics Statement

The studies involving human participants were reviewed and approved by Ethical Committee of Iran University of Medical Sciences. The patients/participants provided their written informed consent to participate in this study.

## Author Contributions

AhH and FT have contributed in conducting the research. MZ has performed the data analysis. AhH, MA, FT, AmH, and MZ have contributed to inscribing the main body of the article. All authors contributed to the article and approved the submitted version.

## Conflict of Interest

The authors declare that the research was conducted in the absence of any commercial or financial relationships that could be construed as a potential conflict of interest.

## Publisher's Note

All claims expressed in this article are solely those of the authors and do not necessarily represent those of their affiliated organizations, or those of the publisher, the editors and the reviewers. Any product that may be evaluated in this article, or claim that may be made by its manufacturer, is not guaranteed or endorsed by the publisher.
